# Disseminated Mycobacterium avium Intracellulare Infection With Concurrent Small Bowel Obstruction: Case, Pathophysiology, and Clinical Considerations

**DOI:** 10.7759/cureus.13469

**Published:** 2021-02-21

**Authors:** Ahjay Bhatia, Himadri Shah, Divy Mehra, Oluwaseun Ogunjemilusi

**Affiliations:** 1 Osteopathic Medicine, Nova Southeastern University Dr. Kiran C. Patel College of Osteopathic Medicine, Davie, USA; 2 Ophthalmology, Nova Southeastern University Dr. Kiran C. Patel College of Osteopathic Medicine, Davie, USA; 3 Internal Medicine, Westside Regional Medical Center, Plantation, USA

**Keywords:** small bowel obstruction, immunocompromised, gastrointestinal stromal tumor, mycobacterium intracellulare, mycobacterium avium complex

## Abstract

Mycobacterium avium intracellulare (MAI) is an opportunistic infection that typically manifests itself as pulmonary infection. In immunocompromised patients, however, MAI can uncommonly cause disseminated disease and diffuse gastrointestinal involvement. Small bowel obstruction with concurrent MAI infection is rarely documented in literature. Here, a 60-year-old female with a past medical history significant for a gastrointestinal stromal tumor, two small bowel obstructions, and a bowel perforation repair presented to the emergency department with sharp abdominal pain due to a small bowel obstruction. Cultures obtained from the laparoscopic release of small bowel obstruction confirmed the presence of MAI. An antibiotic course of ethambutol, azithromycin, and rifampin was initiated and continued upon transfer to a long-term acute care facility. We describe this case to highlight the possibility of MAI infection in patients with postoperative abdominal pain resulting from small bowel obstruction, review the underlying pathophysiology, and discuss its epidemiology.

## Introduction

Mycobacterium avium intracellulare (MAI) is one of two pathogens responsible for nontuberculous mycobacterial infections (Mycobacterium avium complex or MAC). MAI is an opportunistic infection that is commonly seen in immunocompromised (AIDS) patients with low CD4 counts. MAI is found in water, soil, and dust and can be transmitted amongst people through inhalation of respiratory droplets or ingestion into the gastrointestinal tract due to the pathogen’s ability to resist exposure to a pH lower than 3 [1]. MAC is typically seen in immunocompetent patients and manifests itself as a pulmonary infection in those with structural lung disease, however, in AIDS patients the two principal forms of MAC infection are disseminated disease and focal lymphadenitis [2]. Although most commonly seen among AIDS patients with a CD4 count <50 cells/microL, MAI can cause severe infection in other immunocompromised patient populations [2, 3]. Diffuse intestinal involvement is an uncommon manifestation of MAI and small bowel obstruction (SBO) secondary to MAI is sparsely described in literature [4-8]. This case details an immunocompromised patient with an unexpected case of MAI infection with an SBO as a result of diffuse inflammation and dilation. Diagnosis of disseminated MAC infection can be made based on blood cultures and cultures of lymph node cells. CT scans may also be used to monitor disease in other organs. For immunocompromised patients with disseminated or localized infection, a treatment regimen with combination antimicrobial therapy for at least 12 months is recommended [2]. Dual therapy with a macrolide plus ethambutol is common, and a third agent (e.g., rifampin) is added for patients failing antiretroviral therapy (ART) and/or patients with a high mycobacterial burden [2].

## Case presentation

**Figure 1:**
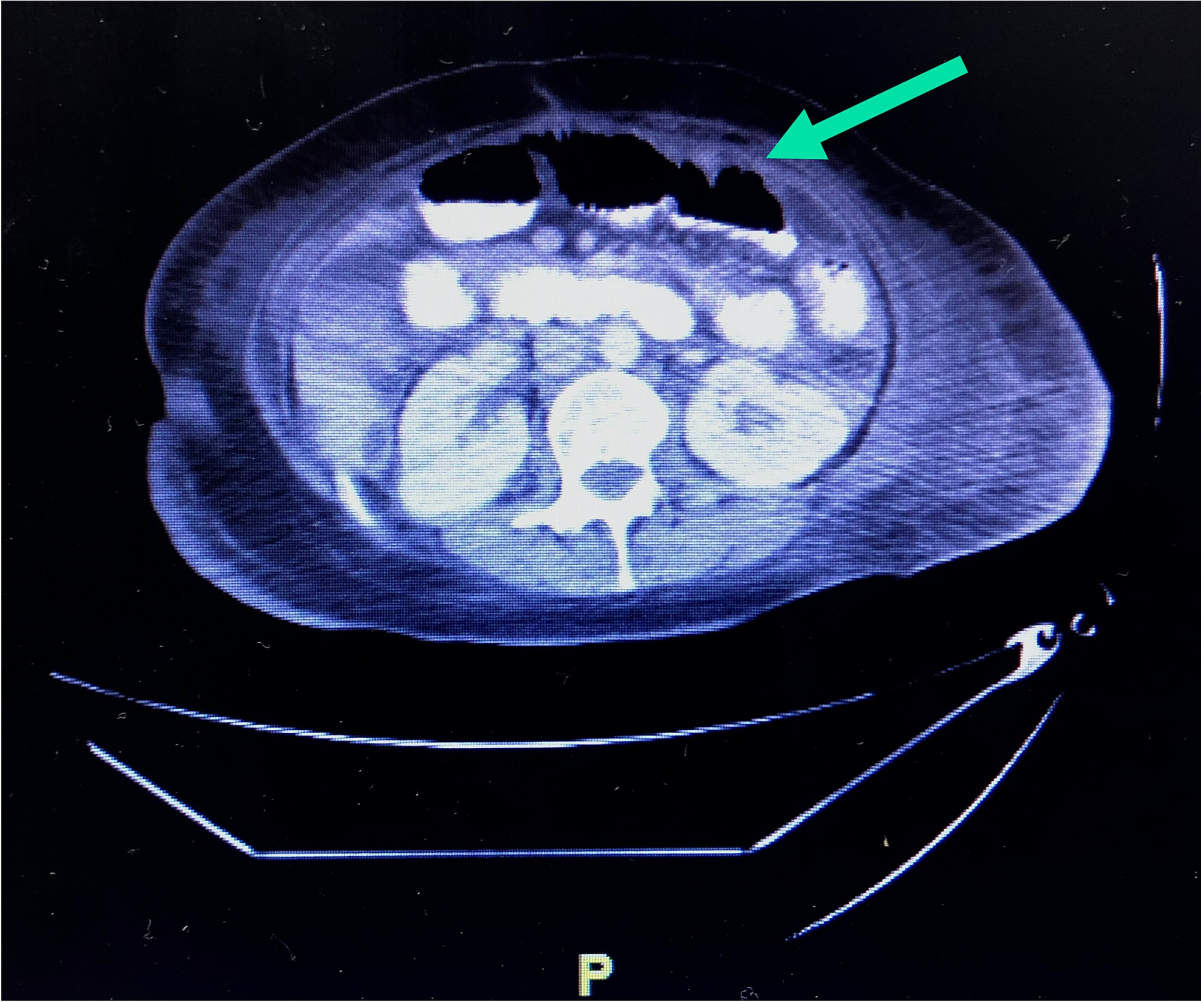
Abdominal computed tomography scan demonstrating diffuse dilation of the small bowel with a focal transition point in the lower quadrant of the abdomen, findings compatible for a worsening high-grade distal small bowel obstruction. Bowel contrast was noted within the proximal and mid small bowel with mild mucosal thickening and decompressed colon.

We report a case of a 60-year-old female with a past medical history significant for gastrointestinal stromal tumor (GIST), perforated bowel, two small bowel obstructions, and most recently, a laparoscopic repair of small bowel perforation, who presented post-operatively to our internal medicine service with abdominal pain. The patient reported approximately five to six days of generalized weakness, abdominal pain, and worsening nausea with episodes of vomiting. The abdominal pain was described as sharp, constant, 10/10 pain that was made worse with vomiting and improved when leaning forward. On initial presentation, the patient was afebrile (98.7°C), tachycardic (129 bpm) and hypotensive (initial reading of 86/56, with a blood pressure range 80-90/50-60 mmHg). Her abdomen was distended, tender to palpation, and tympanic to percussion on physical exam. White blood cell (WBC) was low at 2.6 x 10^3^/µl, which was attributed to a two-year course of sunitinib for previous history of GIST. Initial computed tomography (CT) scan of the abdomen and pelvis revealed diffuse dilation of the small bowel with a focal transition point in the lower quadrant of the abdomen, concerning for a small bowel obstruction. Repeat CT revealed internal worsening with increased small bowel dilatation with intraluminal contrast and no contrast seen within the distal ileum or the colon (Figure 1). Mucosal thickening of the small bowel was identified. A laparoscopic adhesiolysis of the distal jejunum and proximal ileum was performed and an intraoperative surgical culture was taken at that time. The culture returned positive and growth of MAI was detected and isolated during the visit. An antibiotic course of ethambutol, azithromycin, and rifampin was started and continued upon transfer to a long-term acute care facility. Care was then continued in the outpatient setting.

## Discussion

We describe this case to highlight the possibility of MAC infection in patients with postoperative abdominal pain resulting from small bowel obstruction, discussing the epidemiology, pathophysiology, clinical considerations, and treatment approach in such cases. Regarding pathophysiology, we propose that localized MAI-induced inflammation triggered a small bowel obstruction in an individual with a complex abdominal surgical history, as determined by intraoperative culture and isolation. MAC infections found in extrapulmonary settings are uncommon, though there have been a few documented cases in the literature citing intestinal MAC involvement, largely in the context of advanced HIV disease.

Prior to the HIV epidemic, MAC was an unusual source of disease in humans, with rare associations to mild pulmonary infections in individuals with chronic obstructive pulmonary disease (COPD) in addition to lymphadenitis, skin and soft tissue infections, and bone/joint involvement [4-6]. Disseminated infections were even more rare, with just 30 cases documented in the literature prior to 1980, limited to individuals with severe underlying malignancies and immunodeficiencies. Following the rise of HIV and AIDS, MAC became the most common cause of systemic bacterial infection in AIDS patients, and a frequent source of hepatobiliary disease.

MAC (referring to M. avium and M. intracellulare groups) are slow-growing mycobacteria that are ubiquitous in the environment in areas such as soil, poultry, livestock, and water, among others [6]. Their facultative intracellular nature makes them likely to infect the reticuloendothelial system through macrophages, involving the blood, lymph nodes, liver, spleen, bone marrow, and gastrointestinal tract. One study by Koh et al. demonstrated the CT-mediated identification of lymph node enlargement may be an effective tool for early diagnosis of MAC infection in individuals with HIV and abdominal symptoms [4].

Several studies have demonstrated that some forms of MAC can invade intestinal epithelium both in vitro and in vivo [5]. It is most frequently acquired through environmental exposure and absorption through the gastrointestinal tract, though respiratory exposure is possible as well. When MAC infection causes systemic disease, gut involvement is common, with the duodenum being the most frequently affected area. Gastrointestinal symptoms of MAC include diarrhea, abdominal pain, and malabsorption [6].

Small bowel obstructions are a great cause of morbidity in the United States, most frequently resulting from adhesions (65%), hernias (10%), neoplasms (5%), Crohn’s disease (5%), and others (15%). The classic clinical tetrad of SBO includes abdominal pain, nausea and emesis, abdominal distension, constipation, and obstipation. While an SBO can be managed conservatively, there are a few indications to surgically intervene [7]. As in the current case, surgical treatment was indicated given severity of symptoms and the likelihood of strangulation to develop based on the CT findings and past abdominal surgical history.

Disseminated MAC infection, as in the current case, must be treated readily with a combination antimicrobial therapy; a macrolide with ethambutol is a common treatment approach, and must be continued for at least 12 months in individuals in an immunocompromised state with disseminated or localized infection [9]. A third agent, such as rifampin, may be added in appropriate individuals refractory to dual therapy.

## Conclusions

Mycobacterium intracellulare is a source of disseminated and localized gastrointestinal infection, particularly in individuals that are immunocompromised (e.g., neoplastic conditions, human immunodeficiency virus). In rare occasions, localized inflammation from MAI infection may be superimposed on an acutely developed small bowel obstruction. Treatment must be stepwise and interdisciplinary, first addressing acute infection and small bowel obstruction followed by an extended treatment with combination antimicrobials.
